# Superoxide Scavenging by Capers and Kaempferol, Measured by Hydrodynamic Voltammetry, Shows Kaempferol Synergistic Action with Vitamin C; Density Functional Theory (DFT) Results Support Experimental Kaempferol Catalytic Behavior Similar to Superoxide Dismutases (SODs)

**DOI:** 10.3390/molecules30112346

**Published:** 2025-05-27

**Authors:** Miriam Rossi, Stuart Belli, Paloma Velez, Alessio Caruso, Camilla Morresi, Tiziana Bacchetti, Francesco Caruso

**Affiliations:** 1 Department of Chemistry, Vassar College, Poughkeepsie, NY 12604, USA; 2 Department of Chemistry and Chemical Biology, Harvard University, 12 Oxford St., Cambridge, MA 02138, USA; 3 Department of Life and Environmental Sciences, Polytechnic University of Marche, Via Brecce Bianche, 60131 Ancona, Italy

**Keywords:** kaempferol, capers, voltammetry, superoxide, DFT, synergy

## Abstract

In this work, we measured the antioxidant capacity of capers (*Capparis spinosa* L.) and an important component, kaempferol, as scavengers of the superoxide radical anion using hydrodynamic voltammetry with a rotating ring disk electrode (RRDE). Comparing our electrochemical results to other natural products studied using this method, this work demonstrates that kaempferol is a stronger antioxidant than vitamin C, whereas caper extract has weaker antioxidant capability than olive oil. We also investigated the synergistic scavenging relationship between vitamin C and kaempferol and found it to be potent, as all the available superoxide radicals were consumed in the presence of both compounds. Such a dramatic RRDE result was observed for the first time in our laboratory. We then utilized computational Density Functional Theory (DFT) methods to establish a viable mechanism, reminiscent of that exhibited by superoxide dismutase (SOD) enzymes, for the scavenging behavior of kaempferol. In the enzymatic reaction, two molecules of the superoxide radical anion with the assistance of two protons are disproportioned into one molecule of hydrogen peroxide and one oxygen molecule. Our DFT results show kaempferol mimicking superoxide dismutase (SOD) action when one kaempferol molecule reacts with two superoxide radicals and two protons (which can be provided by ascorbic acid); i.e., kaempferol acts as a catalyst that is restored after a cycle of superoxide scavenging. This mechanism is consistent with our experimental RRDE results.

## 1. Introduction

Oxidative stress, caused by the presence of excessive free radicals, can contribute to many disease states and influence healthy aging. Ordinarily, the body has defense mechanisms to combat this stress, which include enzymatic antioxidants such as superoxide dismutases (SODs) and non-enzymatic small-molecule antioxidants such as vitamins E and C. This process is aided by nutritionally obtained antioxidants (mostly polyphenolic compounds). Since oxidative stress is caused by free radical damage to cells and biomolecules, understanding the chemical mechanism by which antioxidants in foods scavenge free radicals is of interest.

The *Capparis spinosa* L. plant is native to the area surrounding the Mediterranean Sea. It grows as a perennial spiny shrub with thick leaves and showy white to pinkish-white flowers, and it thrives under dry conditions. Additionally, it is drought- and salt-tolerant, making it useful for the environment. Various parts of the plant are used in cooking and traditional medicine treatments in areas where it is naturally found [1,2,3,4,5]. Capers are edible flower buds that are fermented and salted. The composition of these flower buds shows capers contain many vitamins, including vitamin C [6]. They also have a significant content of polyphenolic compounds including the flavonoids quercetin (3,5,7,3′,4′-pentahydroxyflavone) and kaempferol (3,5,7,4′-tetrahydroxyflavone) [7]. In fact, capers provide close to 4 mg/g-caper of quercetin and kaempferol [8]. Kaempferol has been the subject of less research than quercetin despite its presence in many edible and medicinal plants such as *Camelia sinensis* (tea). Kaempferol offers many health benefits [9,10], including a decreased cancer risk [11], and is beneficial in breast cancer management [12]. Due to its role in lipid metabolism and inhibiting oxidative stress, kaempferol is useful as a therapeutic agent for the common nonalcoholic [13] and alcoholic [14] liver diseases. Recent reviews describe its effectiveness as an antiviral agent [15], in the treatment of neurodegenerative diseases [16], and as an anti-inflammatory agent and an antioxidant [10,17]. Furthermore, studies describing kaempferol’s effectiveness (in vivo and in vitro) against osteoporosis [18], atherosclerosis [19], diabetes [20], and inflammation [21] exist in the literature. Kaempferol’s effects on neurologic diseases such as Alzheimer’s, Parkinson, ischemia stroke, epilepsy, major depressive disorder, anxiety disorders, neuropathic pain, and even the untreatable glioblastoma tumor have been recently reviewed [22].

Many of the ailments described above are linked to oxidative stress and associated with damage to biological macromolecules such as DNA and proteins caused by excessive concentration of the superoxide radical. In this work, our aim was to measure the antioxidant capacity of caper extract and kaempferol and to clarify the mechanism by which kaempferol acts as an antioxidant to prevent oxidative stress. Earlier studies in our laboratory focused on a hydrodynamic voltammetry method using a rotating ring disk electrode (RRDE) to experimentally measure the scavenging of the important superoxide free radical by whole foods such as anise [23], teas [24], and olive oil [25], along with some of their polyphenolic components. This experimental method has several advantages when studying the short-lived and highly reactive superoxide radical, starting with the generation of the superoxide radical in situ from molecular oxygen by electrochemical means. In our earlier studies, we saw that the examined nutritional compounds had diverse superoxide scavenging mechanisms and, together with the results of this study, we hoped to gain some clarity on this matter. We also investigated the synergistic scavenging relationship between vitamin C and kaempferol, since, in a previous study, we saw markedly increased antioxidant activity when ascorbic acid, vitamin C, was added to the solution of the antioxidant vitamin E [26]. These cooperative relationships support the concept that there are, in many cases, adjuvant plant substances (such as vitamins) which augment the activity of the principal active components responsible for the biochemical outcome [27,28]. We then utilized computational DFT methods to establish a viable mechanism, suggestive of that exhibited by the SOD metalloenzymes, for the scavenging behavior of kaempferol. In that reaction, two molecules of the superoxide radical anion with the assistance of two protons are disproportioned into one molecule of hydrogen peroxide and one of molecular oxygen [29].

## 2. Results and Discussion

### 2.1. RRDE

To measure the antioxidant activity of kaempferol, vitamin C, and capers, we utilized the hydrodynamic voltammetry RRDE method developed in our laboratory, which generates the free radical of interest in situ and allows easy quantification of the reaction between antioxidant and superoxide depletion [30]. Hydrodynamic voltammetry uses convection to enhance the rate of superoxide mass transport to the electrode. The superoxide radical is generated at the disk electrode, and it is dragged by laminar flow to the ring electrode surface where it is destroyed due to its positive potential, 0.25 Volts. However, along the path, superoxide can react with the antioxidant, and so for each antioxidant aliquot added to the voltaic cell, less superoxide is measured at the ring electrode.

#### 2.1.1. Kaempferol

**Figure 1:**
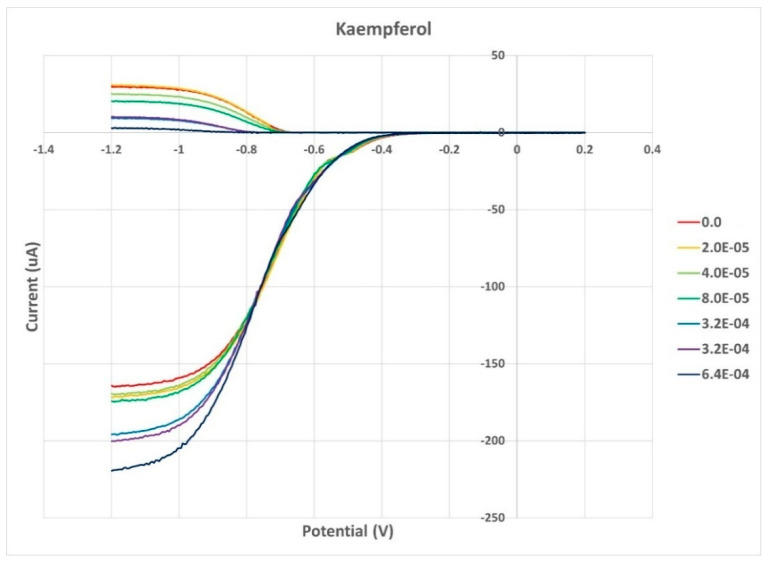
Kaempferol voltammograms indicating the volume of aliquots in liters. For instance, the first aliquot of 20 µL (yellow) is denoted as 2.0 × 10^−5^ (L).

**Figure 2:**
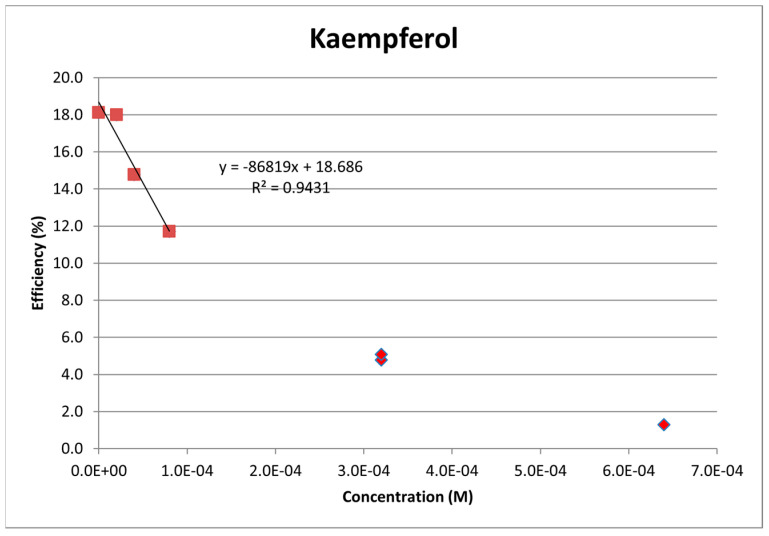
Kaempferol collection efficiency, with slope = −8.7 × 10^4^ M^−1^ calculated from the initial four data points, is lower than that of butein (−11.2 × 10^4^ M^−1^) [31].

**Figure 3:**
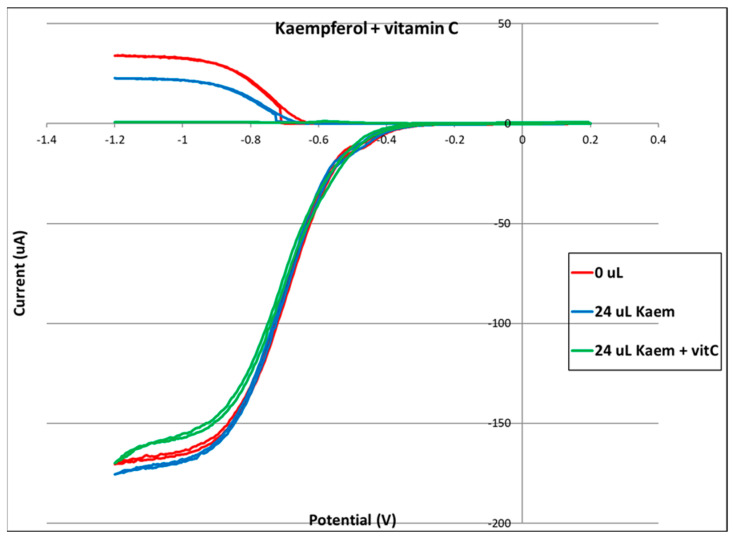
Voltammograms show (1) a blank experiment (red), (2) decreasing superoxide after adding an aliquot (24 µL) of kaempferol (blue), and (3) depletion of superoxide (green) after adding an aliquot of vitamin C (40 µL).

Kaempferol was completely dissolved in DMSO (0.05 M), and aliquots of this solution were added to the electrolytic cell as described in Section 3.4. The resulting voltammograms are shown in Figure 1, and the related collection efficiency is displayed in Figure 2. The initial linear segment of Figure 2 describes the antioxidant property of kaempferol through a slope of −8.7 × 10^4^ M^−1^, which, for comparison with earlier studies, is slightly weaker than that of the chalcone butein (−11.2 × 10^4^ M^−1^) [31] and stronger than that of vitamin C (−2.6 × 10^4^ M^−1^) [26]. The antioxidant activity of vitamin C, both individually and when combined with vitamin E, was recently described by using the DFT and RRDE methods [26]. It was shown that the acidic proton of ascorbic acid reinforced the antioxidant capability of vitamin E. Therefore, we also explore the combination of vitamin C and kaempferol on the scavenging of the superoxide anion using RRDE. Figure 3 summarizes the voltammograms obtained by an individual experiment for kaempferol (24 µL aliquot) and for a 1:1 concentration kaempferol/vitamin C solution, made after adding 40 µL aliquot of 0.03 M vitamin C to the same electrolytic cell. The result of this added vitamin C is dramatic: no superoxide is detected, clearly indicating that there is a synergistic action between these two antioxidants.

From an earlier vitamin C–vitamin E study [26], it was clear that adding only vitamin C to the superoxide solution [10, 20, 40 µL aliquots] resulted in a minimal decrease in superoxide concentration. This is in marked contrast to the results of this study, where we observe complete annihilation of superoxide on adding 40 µL vitamin C to the kaempferol aliquot, as shown in Figure 3. This dramatic result is observed for the first time in our laboratory. We conclude that there is synergistic action when vitamin C and kaempferol scavenge superoxide. Additional investigation for vitamin C/kaempferol action will be described in Section 2.2.

#### 2.1.2. Caper Extract, *C. spinosa*

**Figure 4:**
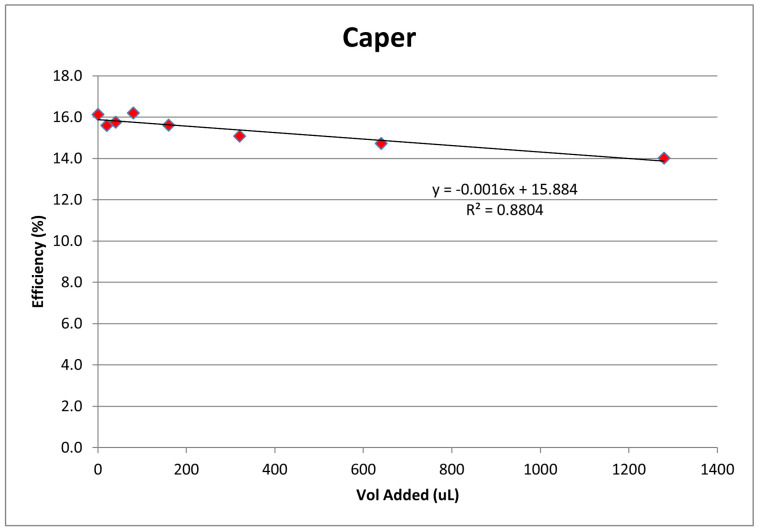
Collection efficiency for DMSO caper extract aliquots of 20, 40, 80, 160, 320, 640, and 1280 µL.

Capers are natural products containing kaempferol. Prior measurements of caper antioxidant activity, which utilize the versatile and widely used DPPH and other assays, are outlined in a recent review [3]. However, our RRDE hydrodynamic voltammetry method allows for more accurate detection of the short-lived superoxide radical due to the constant movement of the solution which promotes a more effective mass transfer to the electrode surface. The extracted supernatant of the caper–DMSO solution was analyzed using the RRDE method. Figure 4 shows the collection efficiency for seven aliquots. When comparing the resulting slope, −1.6 × 10^3^ M^−1^, to other natural products we have studied, we see that it is weaker than that of extra virgin olive oil, −8.3 × 10^2^ M^−1^ [25].

### 2.2. DFT

**Figure 5:**
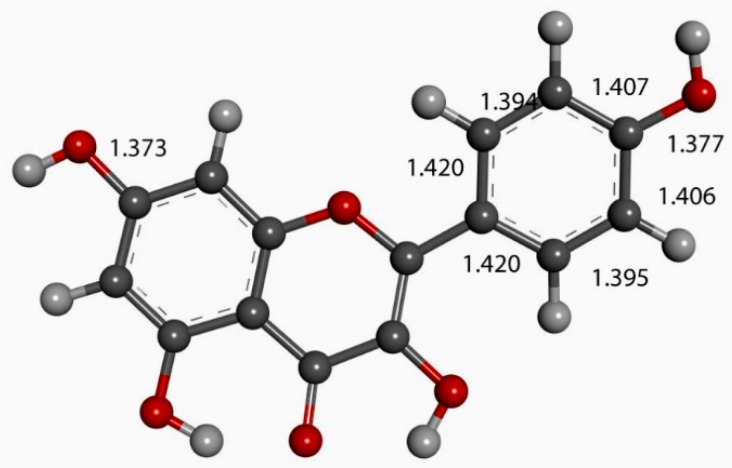
DFT energy-optimized kaempferol molecule. C-C bonds in ring B will be later compared with a related derivative.

To obtain the starting atomic coordinates for the kaempferol molecule, we removed the 3′-hydroxyl group from the closely related quercetin, since we had the X-ray coordinates of quercetin on hand from prior studies. Figure 5 shows the most stable molecular structure obtained after geometry optimization using Dassault Systèmes Biovia software Materials Studio DMOL^3^ [32]. Kaempferol was co-crystallized with propylthiouracil, and, in the crystal structure, the kaempferol molecule was quite flat, with a 4° torsion angle between rings B and C [33]. This experimental result agrees well with the optimized geometry that we use in our calculations, as shown in Figure 5. The kaempferol/propylthiouracil co-crystallization results also demonstrate strong intermolecular interactions whereby the kaempferol molecule shows a strong O-H7 …O4′ hydrogen bond to form a 3-D hydrogen-bonded network. Additionally, a π-π stacking between primarily planar kaempferol molecules is evident (3.38 Å) [33]. These interactions are seen to be important in the DFT results described below.

**Figure 6:**
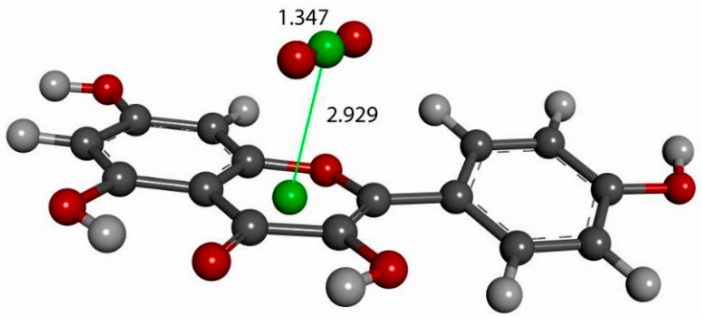
Superoxide approaches kaempferol above the C pyrone ring (van der Waals original separation of 3.50 Å) and establishes a π-π bond, 2.929 Å (green centroids).

Recent studies by our lab demonstrated that scavenging the superoxide radical by polyphenols also involves a π-π approach whereby superoxide stacks on top of aromatic rings [34]. In this study, we explore such an approach on the aromatic C ring of kaempferol, where the initial state has a superoxide radical anion placed above the ring centroid at slightly greater than van der Waals separation, 3.50 Å (the graphite π-π crystal stacking distance is 3.4 Å), to ensure that the placement of superoxide over the ring was an attractive interaction. Upon geometry optimization of the stacked kaempferol–superoxide complex, the resulting energy-minimized product shows a shortened separation between centroids, 2.929 Å, indicating an attractive interaction, as shown in Figure 6.

**Figure 7:**
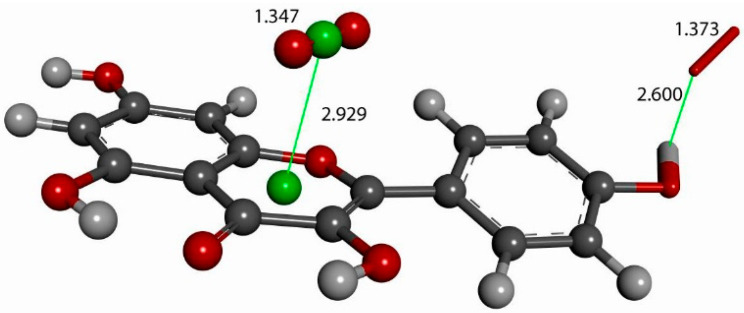
Structural complex resulting from the addition of a second superoxide, via a σ approach to the Figure 6 arrangement, at H4′ (**upper right**, capped stick), with an initial van der Waals separation of 2.60 Å.

**Figure 8:**
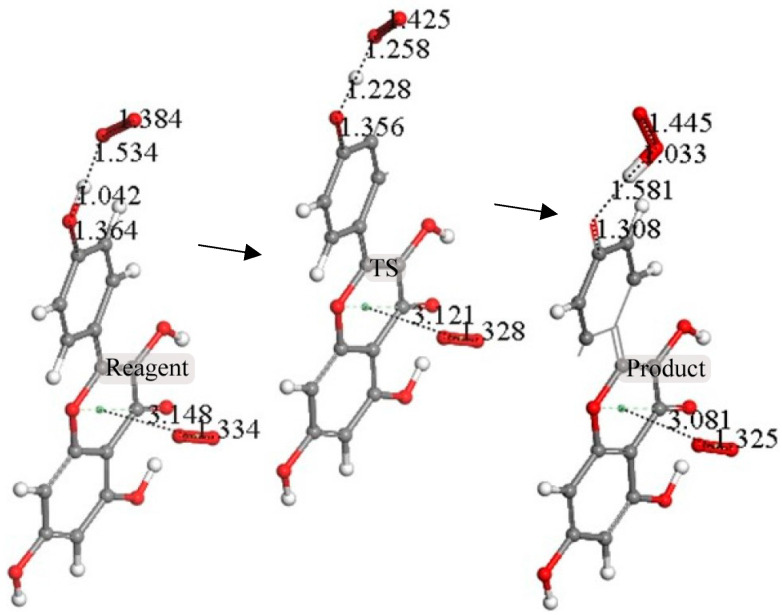
Reaction sequence of H4′ capture by superoxide: optimized reagent, transition state, and product are shown from left to right. ΔG = −0.6 kcal/mol; E(barrier) = 1.6 kcal/mol. The product complex (**right**) shows the formation of an HO_2_ species (**upper right**, capped stick), separated by 1.581 Å from the rest.

**Figure 9:**
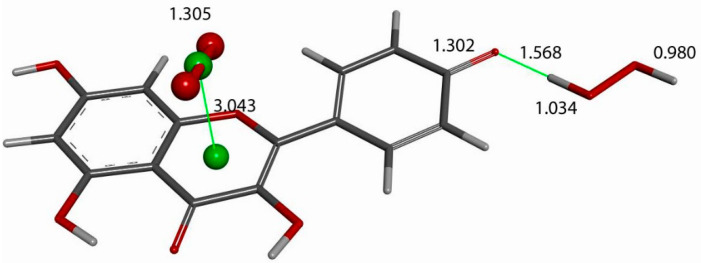
The product from the Figure 8 arrangement is approached by a proton that, upon DFT optimization, binds to the HO_2_ moiety, 0.980 Å, while H_2_O_2_ is formed (**right**), separated by a hydrogen bond, with a distance of 1.568 Å, from the remaining non-radical monoanionic η-O_2_-Kaempferol complex. The original π-π-inserted superoxide (ball and stick) is bound to the C ring, as shown by the centroid–centroid (centroids are shown as green spheres) distance of 3.043 Å.

**Figure 10:**
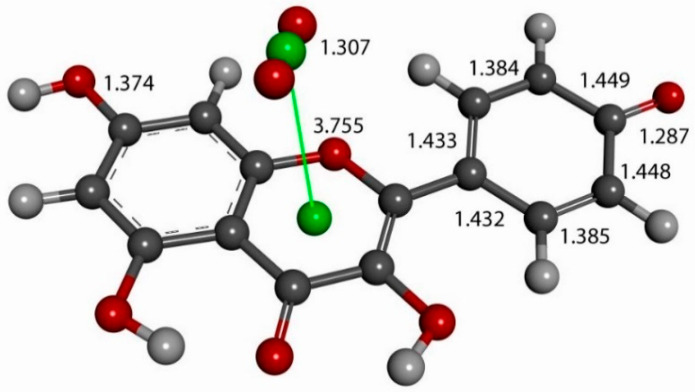
After excluding H_2_O_2_ from the Figure 9 arrangement, DFT optimization shows the π-π-bound superoxide molecule becoming an O_2_ leaving group; i.e., both centroids (green spheres) become separated by 3.755 Å. The attached phenyl ring B shows two C-C bond short lengths, C′2-C′3 (1.385 Å) and C′5-C6′ (1.384 Å), and four elongated lengths, within the range of 1.43—1.45 Å; i.e., there is double–single C-C bond conjugation, consistent with the short C4′-O4′ bond length (1.287 Å). This is in contrast with aromatic C-C bonds seen in Kaempferol, within the range of 1.39–1.41 Å, as shown in Figure 5.

**Figure 11:**
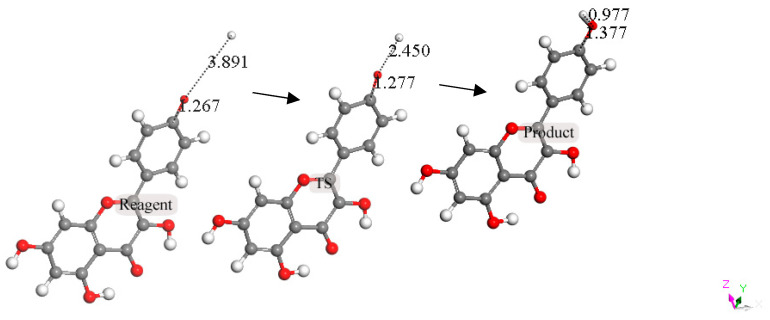
Reaction sequence (reagent, TS, and product), from left to right, for the interaction between O4′ of the kaempferol anion, shown in Figure 10, and a proton. The product on the right shows a COH bond and kaempferol reformation.

The next calculation involved placing a second superoxide at 2.60 Å, the van der Waals distance from the H(hydroxyl) at position 4′ [35]; we refer to this as a σ approach, in contrast with the above-described π approach. Figure 7 shows the initial state of this σ approach. DFT geometrical optimization for this arrangement involves a transition state and the product having H4′ captured by superoxide to form a HO_2_ species. Figure 8 shows the initial- and final-state structures corresponding to the energy minimum. The product indicates the result of geometry optimization obtained after approaching an optimized kaempferol (H4′ excluded) radical separated by 2.60 Å from the HO_2_ species. The ΔG value for this theoretical reaction is −0.6 kcal/mol, and its corresponding E(barrier), 1.6 kcal/mol, is energetically feasible. The formed HO_2_ species is separated by a hydrogen bond from the semiquinone kaempferol moiety, 1.581 Å, indicating the initial O4′-H4′ bond distance, about 1.00 Å, has been broken. Figure S1 shows the corresponding transition state search calculation. The vibrational analysis of the TS structure includes an imaginary frequency at −331 cm^−1^, associated with a first-order saddle point in the energy profile. Later, the most exposed oxygen atom in HO_2_, as shown on the right side of Figure 8, was approached by a proton at a van der Waals distance of 2.60 Å. Upon DFT optimization, this proton was captured (O-H distance = 0.980 Å) and H_2_O_2_ was formed, separated by 1.568 Å from the remaining non-radical η-O_2_-Kaempferol complex (charged-1, due to two negative superoxide radicals and one proton), as shown in Figure 9. H_2_O_2_ was therefore excluded from the arrangement in Figure 9, and the resulting DFT-optimized assembly shows the π-π-bound superoxide molecule, originally inserted in Figure 6, becoming an O_2_ leaving group; i.e., the distance between both centroids increases to 3.755 Å, longer than the initial π-π distance of 3.50 Å. Thus, a molecule of O_2_ has been released and the remaining product conserves the negative charge-1. Meanwhile, the attached B ring shows C-C double–single bond conjugation, consistent with the short C4′-O4′ bond length (1.287 Å), which is much shorter than that of the single C7-O7 bond, 1.374 Å, as shown in Figure 10. This is in marked contrast to the more homogeneous aromatic C-C bond lengths seen in the initial kaempferol structure (Figure 5), which fall in the range of 1.39–1.41 Å. Hence, O4′ of the kaempferol derivative anion, shown in Figure 10, interacts with a proton, and DFT optimization establishes a COH bond and kaempferol reformation, as shown in Figure 11. Related Figures S2 and S3 show the results of the transition state search.

**Figure 12:**
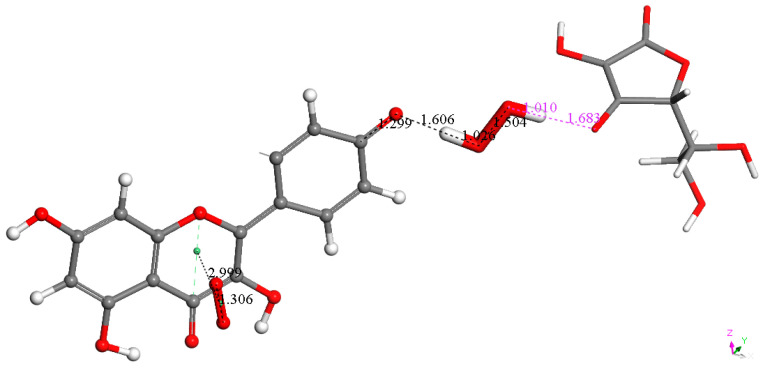
After replacing the reacting proton involved in the Figure 9 arrangement with ascorbic acid, the formation of H_2_O_2_ (center, thick capped stick) is confirmed, and the ascorbate anion (**right**, capped stick) becomes well separated from H_2_O_2_ by 1.683 Å (pink label). The π-π superoxide remains attached to ring C, with a centroid–centroid distance of 2.999 Å, similar to the related bond length of 3.043 Å in Figure 9.

**Figure 13:**
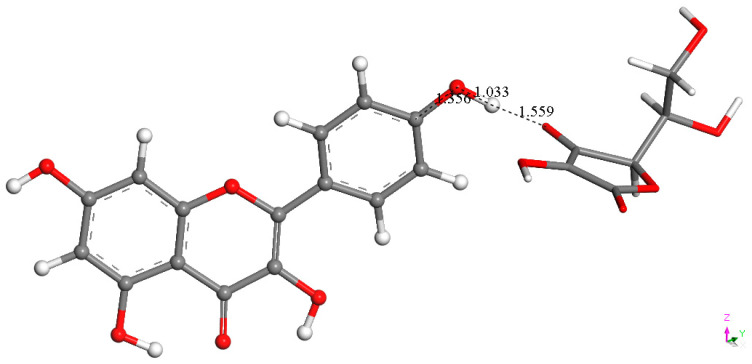
Replacing the reacting proton in Figure 11 with ascorbic acid also shows kaempferol reformation (**left**), well separated from ascorbate (**right**, capped stick), 1.559 Å.

**Figure 14:**
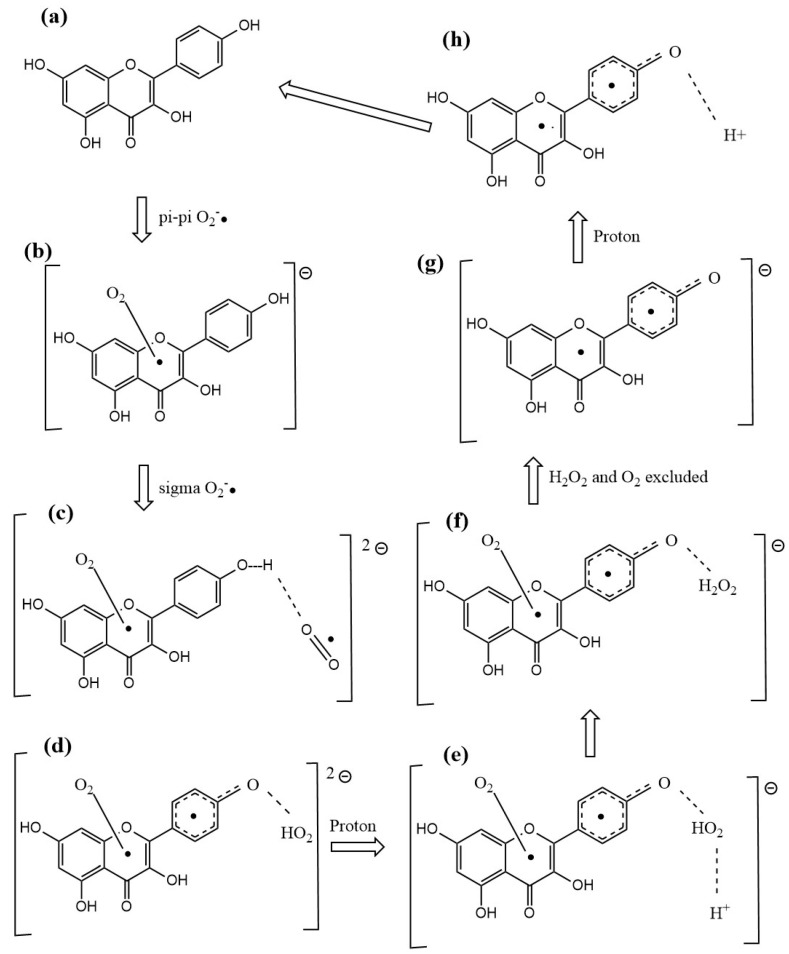
Molecular mechanism for kaempferol scavenging, analogous to that exhibited by the SOD enzymes. (**a**): kaempferol; (**b**): π-π interaction of superoxide with kaempferol, shown in Figure 6 by the short centroid–centroid separation of 2.929 Å; (**c**): van der Waals σ approach of a 2nd superoxide radical; (**d**): HO_2_ formation, indicated by a separation of 1.581 Å (Figure 8, right); (**e**): proton approaching the HO_2_ moiety; (**f**): H_2_O_2_ formation (shown in Figure 9); (**g**): H_2_O_2_ and O_2_ are eliminated; (**h**): a 2nd proton approaches O4′, and OH is formed, restoring kaempferol (Figure 11).

It was also of interest for us to verify if ascorbic acid, considered an important contributor to vitamin E antioxidant activity [26], could play a similar role as a proton donor to kaempferol, shown in Figure 9 and Figure 11. Thus, we replaced the protons described earlier with vitamin C and performed DFT optimizations. Figure 12 and Figure 13 confirm that the vitamin C acidic protons are transferred to the reagents, while the resulting ascorbate anions become well separated, 1.683 Å and 1.559 Å, respectively. We conclude that vitamin C can play a similar role in enhancing kaempferol antioxidant activity as previously seen with vitamin E [26]. More importantly, kaempferol seems to mimic superoxide dismutase (SOD) action, as shown in Equation (1), where the two superoxide radicals are shown in Figure 6 and Figure 7, protons in Figure 9 and Figure 11, molecular oxygen in Figure 10, and hydrogen peroxide in Figure 9. Equation (2) describes the scavenging of superoxide when kaempferol/vitamin C is used. Figure 14 shows the molecular mechanism for kaempferol scavenging that is reminiscent to that exhibited by the SOD enzymes.

2O_2_•^−^ + 2H^+^ → 2O_2_ + H_2_O_2_ (1)

2O_2_•^−^ + 2C_6_H_8_O_6_ (ascorbic acid) → O_2_ + H_2_O_2_ + 2C_6_H_7_O_6_^-^ (ascorbate) (2)

The π-π manner of interaction between antioxidants and superoxide was recently described by our lab and showed varied outcomes [34]. For instance, for the chalcone butein, superoxide remains trapped within the butein ring B, and so an SOD action seems precluded [31]. In contrast, the iso-flavonoid formononetin [36] can release this O_2_ molecule, regenerate itself to become ready for another cycle, and so act as an SOD mimic. In this study, we show that kaempferol behaves as formononetin and that, by including ascorbic acid in the RRDE experiment, complete elimination of superoxide is reached. We conclude that RRDE Figure 3, Section 2.1.1, is consistent with results of the DFT study for a synergistic action between kaempferol and vitamin C.

## 3. Materials and Methods

### 3.1. Materials

Kaempferol was obtained from Indofine Chemical Company (Hillsborough, NJ, USA); tetrabutylammonium bromide (TBAB), 99% anhydrous Dimethyl Sulfoxide (DMSO), and L-Ascorbic acid (vitamin C) were purchased from Sigma-Aldrich (St. Louis, MO, USA).

### 3.2. C. spinosa Subsp. Rupestris Sample Preparation

The *C. spinosa* subsp. rupestris plants were grown in Borgo Cisterna (Santa Lucia Cisterna, Macerata Feltria, PU, Italy). Flower buds of *C. spinosa* were washed, frozen at −20 °C, freeze-dried, and shredded. Flower buds of *C. spinosa* lyophilized (2 g) were processed through ultrasonic-assisted extraction using 0.1% formic acid in 80% methanol as the extraction solvent (1:10 *w*/*v*). The hydro-alcoholic extract has been used for the quantification of total polyphenols and untargeted profiling by ultra-high-performance liquid chromatography–quadrupole time-of-flight mass spectrometry (UHPLC-ESI/qTOF-MS), and the results have been previously published [37]. Earlier quantitative liquid chromatography–quadrupole time-of-flight mass spectrophotometer/mass spectrophotometer (LC-qTOF-MS/MS) analysis [7] and HPLC–DAD analyses of capers confirm the presence of kaempferol and its derivatives in *C. spinosa* [38].

### 3.3. Theoretical Calculations

Theoretical calculations were performed using a molecular modeling program from BIOVIA, Materials Studio 7.0. DMoL^3^ [32], which utilizes Density Functional Theory (DFT) to calculate properties of molecules such as energy, geometry, and transition-state optimizations [39]. We employed the double numerical polarized (DNP) basis set, including all the occupied atomic orbitals plus a second set of valence atomic orbitals, as well as polarized d-valence orbitals [40]. Correlation generalized gradient approximation (GGA) was used, including BLYP correlation and Becke exchange [41]. We also included Grimme’s correction when van der Waals interactions were involved [42]. The continuous model of Dmol^3^ was applied for solvent effects and calculation, including DMSO [43]. All electrons were treated explicitly, and a real-space cutoff of 5 Å was set for the numerical integration of the Hamiltonian matrix elements. The self-consistent field convergence criterion was established such that the root mean square variation in the electronic density was less than 10^−6^ electron/Å^3^. The convergence criteria applied during geometry optimization were 2.72 × 10^−4^ eV for energy and 0.054 eV/Å for force.

### 3.4. RRDE Method

This hydrodynamic voltammetry method has many advantages, including inexpensive instrumentation, small sample sizes, and quick reaction times, making it an ideal method for studying the redox reactions of short-lived molecular species such as the superoxide radical anion. Measuring the antioxidant activity of kaempferol and capers required that they be studied in an electrochemical cell containing 50 mL of 0.1 M TBAB/DMSO solution. TBAB was used as a supporting electrolyte to produce electric current and enhance the occurrence of redox reactions via the gain and loss of electrons (it increases the conductivity of the system). TBAB is stable and does not undergo reduction or oxidation within the potential range of the experiment. Antioxidant activity was measured via the rotating ring disk electrode hydrodynamic voltammetry technique [44] using the WaveDriver 20 bipotentiostat and the MSR Electrode Rotator, from Pine Research, Durham, NC, USA, with a rotating ring disk electrode (RRDE). The main electrode tip was an E6RI ChangeDisk with a rigid gold ring and gold disk (Au/Au) insert. The experimental procedure, before and after each experiment, included using 0.05 µm alumina suspension to clean the main electrode tip. A platinum (Pt) reference electrode and Pt counter electrode from Pine Instrumentation were also used in this experiment. The antioxidant activity of kaempferol and capers was determined individually based on the superoxide radical scavenging ability of each sample that was measured using the protocol developed in our lab [30].

Stock solutions of kaempferol, 0.05 M, and vitamin C, 0.03 M, in anhydrous DMSO were used in trials. Caper stock solution was prepared by adding 0.0506 g of its extract to 10 mL of anhydrous DMSO. This was centrifuged, and the supernatant solution was used for measurements. For the experiment, the electrolytic cell was bubbled for 5 min with a dry O_2_/N_2_ (35%/65%) gas mixture to establish and stabilize its dissolved oxygen level. The Au disk electrode was then rotated at 1000 rpm while the disk was swept from 0.2 V to −1.2 Volts, with the ring held constant at 0.25 Volts. The disk voltage sweep rate was set to 25 mV/s. In summary, runs were performed in the RRDE experiment to determine the antioxidant activity with 20, 40, 80, 160, and 320 aliquots of kaempferol. For the caper sample, runs were performed from 20 to 1280 µL aliquots. An aliquot of 40 μL of 0.03 M vitamin C was added to the kaempferol solution in the electrolytic cell. Results from each run were collected on Aftermath software (Pine Research, Durham, NC, USA) [45] and represented as voltammograms showing current vs. potential graphs that were later analyzed using Microsoft Excel.

In an RRDE voltammetry experiment, the disk electrode is where superoxide radicals are created through the reduction of molecular oxygen (disk current) [O_2_ + e^−^ → O_2_•^−^]. The oxidation of the remaining superoxide radicals (that have not been scavenged by the antioxidant) occurs at the ring electrode. The reverse reaction (ring current) is represented as [O_2_•^−^ → O_2_ + e^−^]. Thus, the rate at which increasing concentrations of kaempferol or caper solution scavenged the generated superoxide radicals during the electrolytic reaction was determined by obtaining the percent value of the quotient of the ring current and the disk current at each concentration. These individual efficiency data were collected using Microsoft Excel and plotted against the corresponding volumes of added kaempferol or caper solution to produce graphs illustrating the effect of increasing concentrations of samples on the scavenging of superoxide radicals in the electrolytic solution. Ultimately, the slope of the curves [30] served as a quantitative measure of the antioxidant activity of kaempferol and capers.

## 4. Conclusions

Reducing the oxidative stress caused by excess free radicals remains an important objective for improving human health, as doing so alleviates damage to the body and reduces the associated risk of disease development [46]. Diet is capable of supplementing endogenous antioxidant activity, including that provided by superoxide dismutase (SOD) enzymes, and can help combat oxidative stress. Understanding the mechanism by which foods and their individual component molecules decrease free radical concentration is an important research objective. In this article, we described the results of experimental antioxidant measurements on capers and an important component, kaempferol, using the RRDE technique. We then described the enhanced antioxidant activity that followed with the addition of vitamin C to kaempferol, resulting in the annihilation of superoxide concentration and suggesting a synergistic relationship between the two molecules. Since capers contain both kaempferol and vitamin C, it is interesting to observe this collaborative association in our experimental studies. We utilized computational DFT methods, whose outcomes support the experimental RRDE results, and arrive at a viable molecular mechanism for kaempferol scavenging that is reminiscent to that exhibited by SOD enzymes. These enzymes catalyze the disproportionation of superoxide radicals (O_2_•^−^) into molecular oxygen (O_2_) and hydrogen peroxide (H_2_O_2_) through a two-step process and essentially neutralize the harmful superoxide radical anions [29]. For SOD enzymes, the reaction is supported by an active site metal ion which interconverts between its oxidized and reduced states. Instead, we see that kaempferol and other plant-based polyphenolic antioxidants act as SOD mimics through suitable intermolecular interactions such as π-π stacking and hydrogen bonding [34,36]. Although capers are widely used in cooking and in traditional medicine formulations, there are few clinical studies on the use of capers and their effect on human health [3,4]. Our findings support the view that there is synergy among different plant components and that this enhances their activity [27]. This work promotes the further exploration of other natural products for potential therapeutic use as free radical scavengers and antioxidants.

## Data Availability

Data are available from the authors.

## Supplementary Materials

The following are available online at https://www.mdpi.com/article/10.3390/molecules30112346/s1: Figure S1: TS search associated with Figure 4; Figure S2: TS search of kaempferol reformation associated with Figure 7. Energy of reaction = −86.091 kcal/mol. Energy of barrier = 3.3 kcal/mol; Figure S3: The imaginary vibrational frequency corresponding to the TS for kaempferol reformation, Figure 7. Frequency = −331 (1/cm). Data regarding Figures S1 and S2.
