# Supplementary material for: Superoxide Scavenging by Capers and Kaempferol, Measured by Hydrodynamic Voltammetry, Shows Kaempferol Synergistic Action with Vitamin C; Density Functional Theory (DFT) Results Support Experimental Kaempferol Catalytic Behavior Similar to Superoxide Dismutases (SODs)
Source: Molecules. 2025 May 27;30(11):2346. doi: 10.3390/molecules30112346 (PMC12155571; doi:10.3390/molecules30112346)
Supplement: Supplementary file 1 [file molecules-30-02346-s001.zip › molecules-3616210-supplementary.pdf]

Supplementary Material

Superoxide scavenging by capers and kaempferol, measured by hydrodynamic voltammetry, shows synergistic action between kaempferol and vitamin C. Density Functional Theory (DFT) results support experimental kaempferol catalytic behavior similar to superoxide dismutases (SOD)

Miriam Rossi[1],* Stuart Belli[1], Paloma Velez[1], Alessio Caruso[2], Camilla Morresi[3], Tiziana Bacchetti[3], Francesco Caruso[1],*.

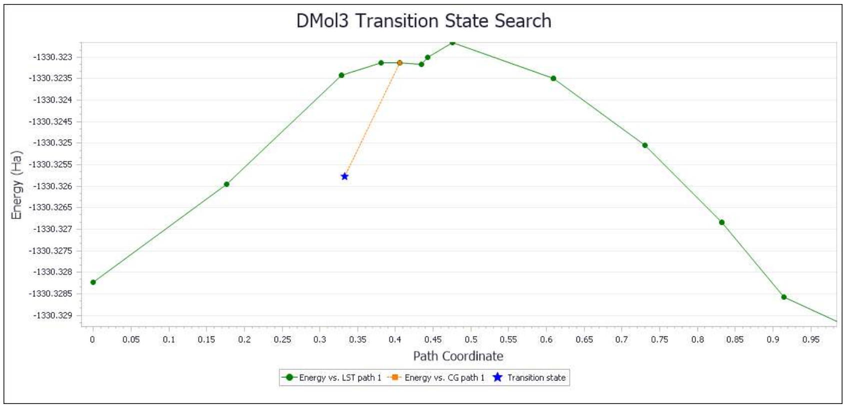

Figure S1. TS Search associated with Figure 8.

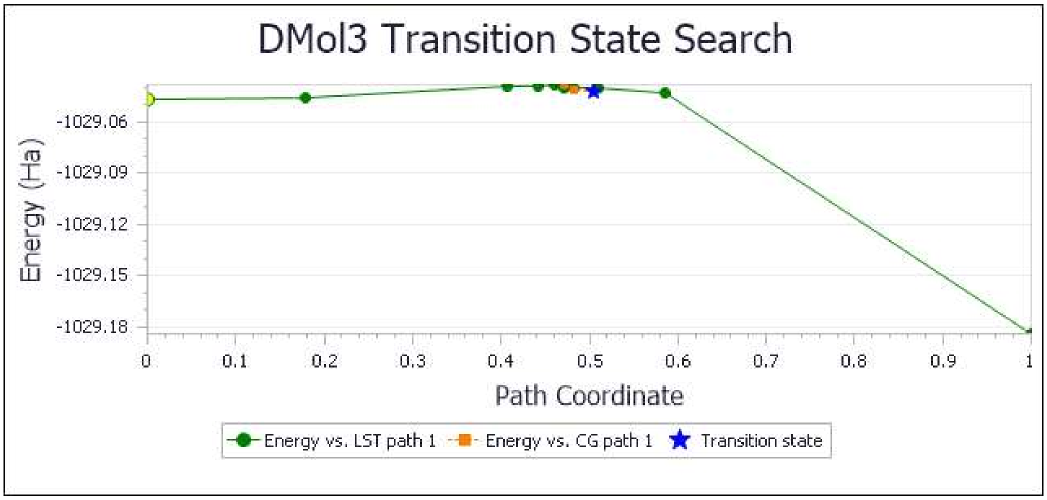

Figure S2. TS search of kaempferol reformation associated with Figure 11. Energy of reaction = -86.091 kcal/mol. Energy of barrier = 3.3 kcal/mol

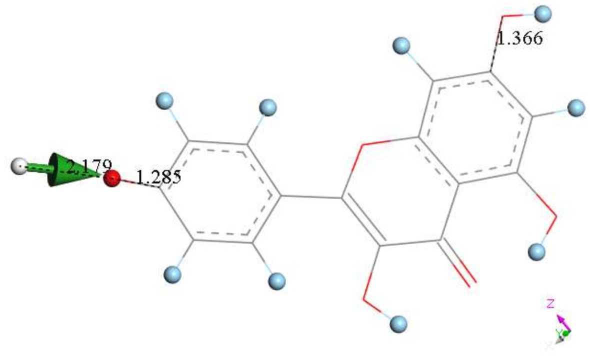

Figure S3. The imaginary vibrational frequency corresponding to the TS for kaempferol reformation, Figure 11. Frequency = -331 (1/cm).

Cartesian coordinates regarding Figure 8

REACTANT

!BIOSYM archive 3

PBC=OFF

Materials Studio Generated CAR File

!DATE Wed Feb 15 12:36:26 2023

O1 -0.963992390 -1.194210202 -3.483183090 XXXX 1 xx O 0.000

O2 -0.845431745 -0.454961926 0.576341087 XXXX 1 xx O 0.000

O3 -4.159854274 -3.701141997 1.558111836 XXXX 1 xx O 0.000

H1 -4.755042246 -4.341913996 1.121413741 XXXX 1 xx H 0.000

O4 -2.817865631 -3.016534306 -3.035157701 XXXX 1 xx O 0.000

H2 -2.155816671 -2.411451629 -3.504252283 XXXX 1 xx H 0.000

O5 0.770926106 0.630055592 -2.547939410 XXXX 1 xx O 0.000

H3 0.527919147 0.229902936 -3.418614786 XXXX 1 xx H 0.000

C1 -1.786210185 -1.708508441 -1.276455848 XXXX 1 xx C 0.000

C2 0.016822016 0.251919842 -0.276162136 XXXX 1 xx C 0.000

C3 -2.487574363 -2.091838737 1.057091518 XXXX 1 xx C 0.000

H4	-2.410091914	-1.851677777	2.115085979	XXXX	1	xx	H	0.000
C4	-1.700355974	-1.428719237	0.108132499	XXXX	1	xx	C	0.000
C5	-3.507435657	-3.391047021	-0.769819560	XXXX	1	xx	C	0.000
H5	-4.208067255	-4.155910992	-1.103325685	XXXX	1	xx	H	0.000
C6	0.870095701	1.178829953	0.443769441	XXXX	1	xx	C	0.000
C7	-3.390012680	-3.068750662	0.598481065	XXXX	1	xx	C	0.000
C8	-0.050821184	-0.010563374	-1.643042024	XXXX	1	xx	C	0.000
C9	-0.945550814	-0.988929332	-2.197270431	XXXX	1	xx	C	0.000
C10	1.639420466	2.183028200	-0.215992052	XXXX	1	xx	C	0.000
H6	1.597904752	2.274157104	-1.295501268	XXXX	1	xx	H	0.000
C11	2.445050209	3.072090956	0.499799563	XXXX	1	xx	C	0.000
C12	2.520048390	3.001940580	1.912033963	XXXX	1	xx	C	0.000
C13	-2.709120819	-2.713506813	-1.701604241	XXXX	1	xx	C	0.000
C14	0.961255971	1.122576354	1.869130159	XXXX	1	xx	C	0.000
H7	0.400238093	0.366940088	2.412680841	XXXX	1	xx	H	0.000
C15	1.766393885	2.010646301	2.584690228	XXXX	1	xx	C	0.000
O6	3.290021815	3.852417668	2.649321452	XXXX	1	xx	O	0.000
H8	1.823075802	1.947543215	3.671521785	XXXX	1	xx	H	0.000
H9	3.022615387	3.834919104	-0.020975418	XXXX	1	xx	H	0.000
H10	3.761330919	4.549809180	2.035886341	XXXX	1	xx	H	0.000
O7	1.637203321	-2.755034832	-1.308582966	XXXX	1	xx	O	0.000
O8	1.608154674	-2.407026923	-0.021094589	XXXX	1	xx	O	0.000
O9	4.437727174	5.626217802	1.177152944	XXXX	1	xx	O	0.000
O10	5.797039973	5.558733325	1.428329044	XXXX	1	xx	O	0.000

end

end

PRODUCT

!BIOSYM archive 3

PBC=OFF

Materials Studio Generated CAR File

!DATE Wed Feb 15 12:36:26 2023

O1	-0.997004215	-1.173657239	-3.490789516	XXXX	1	xx	O	0.000
O2	-0.927846742	-0.368610856	0.548699992	XXXX	1	xx	O	0.000
O3	-4.008051326	-3.812137987	1.589083295	XXXX	1	xx	O	0.000
H1	-4.538647600	-4.517261741	1.168744089	XXXX	1	xx	H	0.000
O4	-2.713008970	-3.124702500	-3.010671313	XXXX	1	xx	O	0.000
H2	-2.086679366	-2.494762357	-3.492325779	XXXX	1	xx	H	0.000
O5	0.670891245	0.720472614	-2.576091019	XXXX	1	xx	O	0.000
H3	0.451176081	0.302536543	-3.444923187	XXXX	1	xx	H	0.000
C1	-1.795194404	-1.700942775	-1.281288567	XXXX	1	xx	C	0.000
C2	-0.081531661	0.351643270	-0.302473960	XXXX	1	xx	C	0.000
C3	-2.463873371	-2.091238852	1.058685360	XXXX	1	xx	C	0.000
H4	-2.391846299	-1.834729220	2.112914611	XXXX	1	xx	H	0.000
C4	-1.724186938	-1.395209446	0.096676031	XXXX	1	xx	C	0.000
C5	-3.388929201	-3.492127957	-0.743633436	XXXX	1	xx	C	0.000
H5	-4.029460312	-4.314165313	-1.060676628	XXXX	1	xx	H	0.000
C6	0.756905574	1.288288123	0.416798197	XXXX	1	xx	C	0.000
C7	-3.292560828	-3.139051164	0.618868800	XXXX	1	xx	C	0.000
C8	-0.137996173	0.071151058	-1.669198027	XXXX	1	xx	C	0.000
C9	-0.991723741	-0.952174930	-2.211787506	XXXX	1	xx	C	0.000
C10	1.566142524	2.270023775	-0.237126926	XXXX	1	xx	C	0.000
H6	1.544550595	2.351000459	-1.318581113	XXXX	1	xx	H	0.000
C11	2.382894786	3.135836991	0.482552403	XXXX	1	xx	C	0.000
C12	2.460617598	3.090262819	1.919500631	XXXX	1	xx	C	0.000
C13	-2.639814373	-2.778472175	-1.687801072	XXXX	1	xx	C	0.000
C14	0.813036131	1.251433172	1.847714472	XXXX	1	xx	C	0.000
H7	0.214322200	0.520286085	2.385350509	XXXX	1	xx	H	0.000

```
C15     1.623324324    2.117302331    2.570350276 XXXX 1     xx     C  0.000
O6      3.239459547    3.886276385    2.605919830 XXXX 1     xx     O  0.000
H8      1.653415623    2.060348508    3.659143413 XXXX 1     xx     H  0.000
H9      2.984213584    3.878571185   -0.042488688 XXXX 1     xx     H  0.000
H10     4.373167669    4.570749717    1.742754301 XXXX 1     xx     H  0.000
O7      1.263847642   -2.948619427   -1.377828592 XXXX 1     xx     O  0.000
O8      1.267706045   -2.553584160   -0.113295186 XXXX 1     xx     O  0.000
O9      5.145939598    4.948182170    1.171295058 XXXX 1     xx     O  0.000
O10     5.796744754    5.877082902    2.065929243 XXXX 1     xx     O  0.000
end
end

TS
!BIOSYM archive 3
PBC=OFF

!DATE     Feb 15 14:56:45 2023
O1     -0.978539332   -1.187007593   -3.486585779 XXXX 1     xx     O  0.000
O2     -0.882606299   -0.415490661    0.562169712 XXXX 1     xx     O  0.000
O3     -4.097230024   -3.748011196    1.570931829 XXXX 1     xx     O  0.000
H1     -4.666275479   -4.416516412    1.143243893 XXXX 1     xx     H  0.000
O4     -2.773894875   -3.066422172   -3.024281211 XXXX 1     xx     O  0.000
H2     -2.127458743   -2.449714970   -3.499775014 XXXX 1     xx     H  0.000
O5      0.729047361    0.666665918   -2.560882659 XXXX 1     xx     O  0.000
H3      0.495666796    0.259379967   -3.430766655 XXXX 1     xx     H  0.000
C1     -1.790241837   -1.706162795   -1.279718203 XXXX 1     xx     C  0.000
C2     -0.026284563    0.292250477   -0.288590630 XXXX 1     xx     C  0.000
C3     -2.478785136   -2.090161033    1.056805863 XXXX 1     xx     C  0.000
H4     -2.403383107   -1.842774176    2.113760902 XXXX 1     xx     H  0.000
```

C4 -1.711370968 -1.413316357 0.103243965 XXXX 1 xx C 0.000

C5 -3.458074552 -3.435215260 -0.758526872 XXXX 1 xx C 0.000

H5 -4.134903307 -4.224958534 -1.083886489 XXXX 1 xx H 0.000

C6 0.825092648 1.223225830 0.432210819 XXXX 1 xx C 0.000

C7 -3.349861472 -3.099602435 0.608744068 XXXX 1 xx C 0.000

C8 -0.087452484 0.024147459 -1.655805495 XXXX 1 xx C 0.000

C9 -0.966708662 -0.973761570 -2.204369885 XXXX 1 xx C 0.000

C10 1.612456543 2.213911246 -0.225810022 XXXX 1 xx C 0.000

H6 1.577149762 2.304655260 -1.305962679 XXXX 1 xx H 0.000

C11 2.429372239 3.087727855 0.492335761 XXXX 1 xx C 0.000

C12 2.500512742 3.036693113 1.917448791 XXXX 1 xx C 0.000

C13 -2.679444777 -2.744113555 -1.695146018 XXXX 1 xx C 0.000

C14 0.899780098 1.173830663 1.859830192 XXXX 1 xx C 0.000

H7 0.322223618 0.428806340 2.399932450 XXXX 1 xx H 0.000

C15 1.709062071 2.047851593 2.575461075 XXXX 1 xx C 0.000

O6 3.301063146 3.880875176 2.614803908 XXXX 1 xx O 0.000

H8 1.750709064 1.990243056 3.666498289 XXXX 1 xx H 0.000

H9 3.031200668 3.828382502 -0.034863512 XXXX 1 xx H 0.000

H10 3.947760809 4.658113311 1.918288046 XXXX 1 xx H 0.000

O7 1.483861888 -2.839478470 -1.337725122 XXXX 1 xx O 0.000

O8 1.467453488 -2.471782269 -0.062202497 XXXX 1 xx O 0.000

O9 4.592604663 5.479111255 1.215651034 XXXX 1 xx O 0.000

O10 5.937498012 5.528618442 1.683538140 XXXX 1 xx O 0.000

end

end

Cartesian coordinates regarding Figure 11

REACTANT

!BIOSYM archive 3

PBC=OFF

Materials Studio Generated CAR File

!DATE Fri Feb 17 14:06:37 2023

O1	-0.253069962	-0.580252140	-3.634189289	XXXX	1	xx	O	0.000
O2	-0.494597343	-0.392373864	0.454650936	XXXX	1	xx	O	0.000
O3	-3.797115839	-3.739648705	0.724337408	XXXX	1	xx	O	0.000
H1	-4.332152224	-4.353003363	0.180803400	XXXX	1	xx	H	0.000
O4	-2.126316670	-2.495073175	-3.595927027	XXXX	1	xx	O	0.000
H2	-1.456037763	-1.849524509	-3.967464793	XXXX	1	xx	H	0.000
O5	1.316014294	1.119923816	-2.320891954	XXXX	1	xx	O	0.000
H3	1.167956647	0.868716018	-3.270041716	XXXX	1	xx	H	0.000
C1	-1.265485864	-1.392672650	-1.618526998	XXXX	1	xx	C	0.000
C2	0.399304089	0.440977822	-0.198062603	XXXX	1	xx	C	0.000
C3	-2.146955683	-2.064841413	0.579247224	XXXX	1	xx	C	0.000
H4	-2.156092364	-1.966651843	1.661472763	XXXX	1	xx	H	0.000
C4	-1.302311093	-1.287489640	-0.207090377	XXXX	1	xx	C	0.000
C5	-2.983068219	-3.143454074	-1.477369875	XXXX	1	xx	C	0.000
H5	-3.636105717	-3.869922277	-1.957493805	XXXX	1	xx	H	0.000
C6	1.160829770	1.268803802	0.696988365	XXXX	1	xx	C	0.000
C7	-2.985016038	-2.993102413	-0.075108852	XXXX	1	xx	C	0.000
C8	0.466258731	0.369203973	-1.599597173	XXXX	1	xx	C	0.000
C9	-0.371456463	-0.560275631	-2.366722092	XXXX	1	xx	C	0.000
C10	1.994213493	2.343442075	0.227606884	XXXX	1	xx	C	0.000
H6	2.052642319	2.557731056	-0.832727917	XXXX	1	xx	H	0.000
C11	2.703441135	3.127516861	1.106085277	XXXX	1	xx	C	0.000
C12	2.649775687	2.899858342	2.548646692	XXXX	1	xx	C	0.000
C13	-2.130452765	-2.350835399	-2.248843046	XXXX	1	xx	C	0.000
C14	1.087342493	1.033844982	2.117115687	XXXX	1	xx	C	0.000

H7 0.465883419 0.224699648 2.490142007 XXXX 1 xx H 0.000

C15 1.799100269 1.800769470 3.005307117 XXXX 1 xx C 0.000

O6 3.302874057 3.617278911 3.363421622 XXXX 1 xx O 0.000

H8 1.752850637 1.606886402 4.075918230 XXXX 1 xx H 0.000

H9 3.321489197 3.948733284 0.746595511 XXXX 1 xx H 0.000

H10 5.796257773 5.810734632 5.391718395 XXXX 1 xx H 0.000

end

end

PRODUCT

!BIOSYM archive 3

PBC=OFF

Materials Studio Generated CAR File

!DATE Fri Feb 17 14:06:37 2023

O1 -0.247882937 -0.544035232 -3.588526603 XXXX 1 xx O 0.000

O2 -0.433418896 -0.292281151 0.496563607 XXXX 1 xx O 0.000

O3 -3.706627320 -3.688698457 0.854843210 XXXX 1 xx O 0.000

H1 -4.223106943 -4.318132059 0.313708168 XXXX 1 xx H 0.000

O4 -2.088040963 -2.443831494 -3.505933058 XXXX 1 xx O 0.000

H2 -1.418220170 -1.782423635 -3.861986617 XXXX 1 xx H 0.000

O5 1.358498634 1.180579550 -2.342700450 XXXX 1 xx O 0.000

H3 1.180159702 0.889244054 -3.271231832 XXXX 1 xx H 0.000

C1 -1.222777576 -1.331208710 -1.542679609 XXXX 1 xx C 0.000

C2 0.455129976 0.520897756 -0.198060912 XXXX 1 xx C 0.000

C3 -2.076642957 -1.986224881 0.672692631 XXXX 1 xx C 0.000

H4 -2.079488116 -1.871878557 1.753349792 XXXX 1 xx H 0.000

C4 -1.246052144 -1.207230397 -0.134625676 XXXX 1 xx C 0.000

C5 -2.918973388 -3.088939068 -1.361763936 XXXX 1 xx C 0.000

H5 -3.568652064 -3.827449895 -1.829068028 XXXX 1 xx H 0.000

```
C6      1.248189713    1.369146879    0.699681717 XXXX 1     xx     C   0.000
C7     -2.904578757   -2.927267644    0.040455974 XXXX 1     xx     C   0.000
C8      0.504293337    0.427924377   -1.576723292 XXXX 1     xx     C   0.000
C9     -0.338494906   -0.498205502   -2.310463137 XXXX 1     xx     C   0.000
C10     2.080788668    2.414260663    0.219819376 XXXX 1     xx     C   0.000
H6      2.142484857    2.620762630   -0.842556941 XXXX 1     xx     H   0.000
C11     2.827105000    3.202362761    1.095736826 XXXX 1     xx     C   0.000
C12     2.766186330    2.968677285    2.480560193 XXXX 1     xx     C   0.000
C13    -2.085777540   -2.296914456   -2.150935321 XXXX 1     xx     C   0.000
C14     1.198147630    1.157789187    2.102553924 XXXX 1     xx     C   0.000
H7      0.572462221    0.369281794    2.510446165 XXXX 1     xx     H   0.000
C15     1.944671732    1.942377096    2.980715309 XXXX 1     xx     C   0.000
O6      3.526149553    3.774373109    3.298392004 XXXX 1     xx     O   0.000
H8      1.892510396    1.759955740    4.054205787 XXXX 1     xx     H   0.000
H9      3.458898275    4.002502266    0.713785147 XXXX 1     xx     H   0.000
H10     3.403058658    3.504585986    4.229745581 XXXX 1     xx     H   0.000
end
end
```

TS

```
!BIOSYM archive 3
PBC=OFF

!DATE     Feb 17 16:40:28 2023
O1     -0.261824099   -0.574685753   -3.631469306 XXXX 1     xx     O   0.000
O2     -0.435091052   -0.342025998    0.453737120 XXXX 1     xx     O   0.000
O3     -3.722742166   -3.680645515    0.839022501 XXXX 1     xx     O   0.000
H1     -4.260108218   -4.309927890    0.318893492 XXXX 1     xx     H   0.000
O4     -2.126230157   -2.488630518   -3.541720189 XXXX 1     xx     O   0.000
```

H2	-1.458612186	-1.838739137	-3.914632053	XXXX	1	xx	H	0.000
O5	1.349271723	1.133277811	-2.362445020	XXXX	1	xx	O	0.000
H3	1.173933521	0.864341717	-3.301078823	XXXX	1	xx	H	0.000
C1	-1.243446321	-1.365095571	-1.593434120	XXXX	1	xx	C	0.000
C2	0.446241019	0.488684229	-0.221662503	XXXX	1	xx	C	0.000
C3	-2.086429297	-2.007340414	0.627986951	XXXX	1	xx	C	0.000
H4	-2.078399375	-1.891538413	1.709364875	XXXX	1	xx	H	0.000
C4	-1.256620629	-1.240931910	-0.184087012	XXXX	1	xx	C	0.000
C5	-2.955858716	-3.120712665	-1.398565459	XXXX	1	xx	C	0.000
H5	-3.616337963	-3.858922891	-1.850895392	XXXX	1	xx	H	0.000
C6	1.209676757	1.328915845	0.662374989	XXXX	1	xx	C	0.000
C7	-2.931923862	-2.946748002	0.002442924	XXXX	1	xx	C	0.000
C8	0.501238241	0.394886756	-1.619536510	XXXX	1	xx	C	0.000
C9	-0.356853904	-0.538779022	-2.359362970	XXXX	1	xx	C	0.000
C10	2.048900988	2.394386660	0.190615998	XXXX	1	xx	C	0.000
H6	2.083324144	2.613415242	-0.868235161	XXXX	1	xx	H	0.000
C11	2.810643501	3.139443041	1.064725962	XXXX	1	xx	C	0.000
C12	2.718521553	2.963134132	2.510832629	XXXX	1	xx	C	0.000
C13	-2.118059321	-2.334502645	-2.194393127	XXXX	1	xx	C	0.000
C14	1.155299592	1.103421547	2.083168359	XXXX	1	xx	C	0.000
H7	0.513832976	0.319169196	2.469372253	XXXX	1	xx	H	0.000
C15	1.904443150	1.843265603	2.968322981	XXXX	1	xx	C	0.000
O6	3.087108914	3.894012314	3.303419994	XXXX	1	xx	O	0.000
H8	1.815576449	1.676284423	4.038032193	XXXX	1	xx	H	0.000
H9	3.404400093	3.975731125	0.711780044	XXXX	1	xx	H	0.000
H10	4.686124652	4.406856700	5.087424383	XXXX	1	xx	H	0.000

end

end
